# Teaching about the Wild: A Survival Course as a Novel Resident Educational Experience

**DOI:** 10.21980/J8N06R

**Published:** 2021-04-19

**Authors:** Geoffrey Comp, Rachel Munn, Renee Moffitt, Eric Cortez

**Affiliations:** * Valleywise Health Medical Center/Creighton University School of Medicine (Phoenix), Department of Emergency Medicine, Phoenix, AZ; ^ University of Arizona College of Medicine, Department of Emergency Medicine, Tucson, AZ; † Presbyterian Healthcare Services, Department of Emergency Medicine, Albuquerque, NM; ^^ OhioHealth Doctors Hospital/OhioHealth EMS, Department of Emergency Medicine, Columbus, OH

## Abstract

**Audience:**

Emergency medicine residents.

**Introduction:**

Wilderness medicine (WM) is the practice of resource-limited medicine under austere conditions. Emergency physicians in training should gain additional exposure to wilderness medicine knowledge and outdoor skills to allow for the development of problem solving and improvisation abilities. However, there is limited data on the instruction of general survival skills to residents interested in WM.

**Educational Objectives:**

By the end of the session the learner will be able to: 1) differentiate at least three different methods for water purification 2) describe how to erect a temporary survival shelter 3) construct a survival pack for personal use emphasizing multi-use items 4) demonstrate how to make a fire without a direct flame supply.

**Educational Methods:**

A small group of resident learners progressed through five survival stations designed to allow for an emphasis on select skills, wilderness medicine knowledge, and improvisation. Resident instructors led the discussion and skills demonstration.

**Research Methods:**

Participants completed a six item before and after questionnaire. Each item was ranked from 0 for “strongly disagree” to 10 for “strongly agree.” Total mean scores before and after were compared.

**Results:**

Twelve individuals participated. The total mean score for the six-item analysis increased following the workshop (39.1 before versus 51.0 after, p = 0.0008).

**Discussion:**

General survival skills are traditionally acquired through time-intensive experiences; however, this is often unfeasible during residency training. We developed an alternative, more efficient mechanism for incorporating wilderness medicine skills into residency training that appears to improve understanding and confidence of participants, as well as to provide a teaching opportunity for new resident educators.

**Topics:**

Wilderness medicine, survival skills, emergency medicine, graduate medical education.

## USER GUIDE

**Learner Audience:**

Medical Students, Interns, Junior Residents, Senior Residents

**Time Required for Implementation:**

2–3 hours

**Recommended Number of Learners per Instructor:**

8–12

**Topics:**

Wilderness medicine, survival skills, emergency medicine, graduate medical education.

**Objectives:**

By the end of the session the learner will be able to:

1. Differentiate at least three different methods for water purification
2. Describe how to erect a temporary survival shelter
3. Construct a survival pack for personal use emphasizing multi-use items
4. Demonstrate how to make a fire without a direct flame supply
5. Recall the steps to use a compass for basic navigation and orienteering tasks

Furthermore, by the end of the session the resident educator will:

1. Prepare a 30-minute presentation and demonstration of a wilderness survival topic for their peer learners
2. Demonstrate an example of their specific survival skill and teach the other peer learners’ effective application

### Linked objectives, methods and results

**Image 1:**
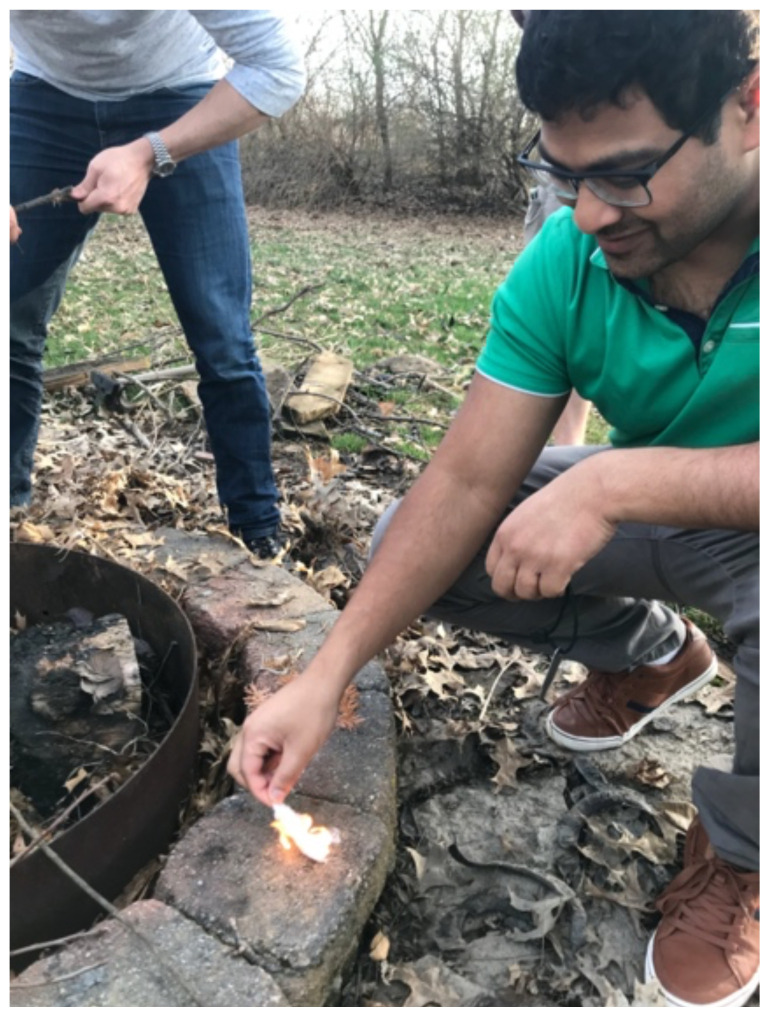
Fire starting. Author’s own image

**Image 2:**
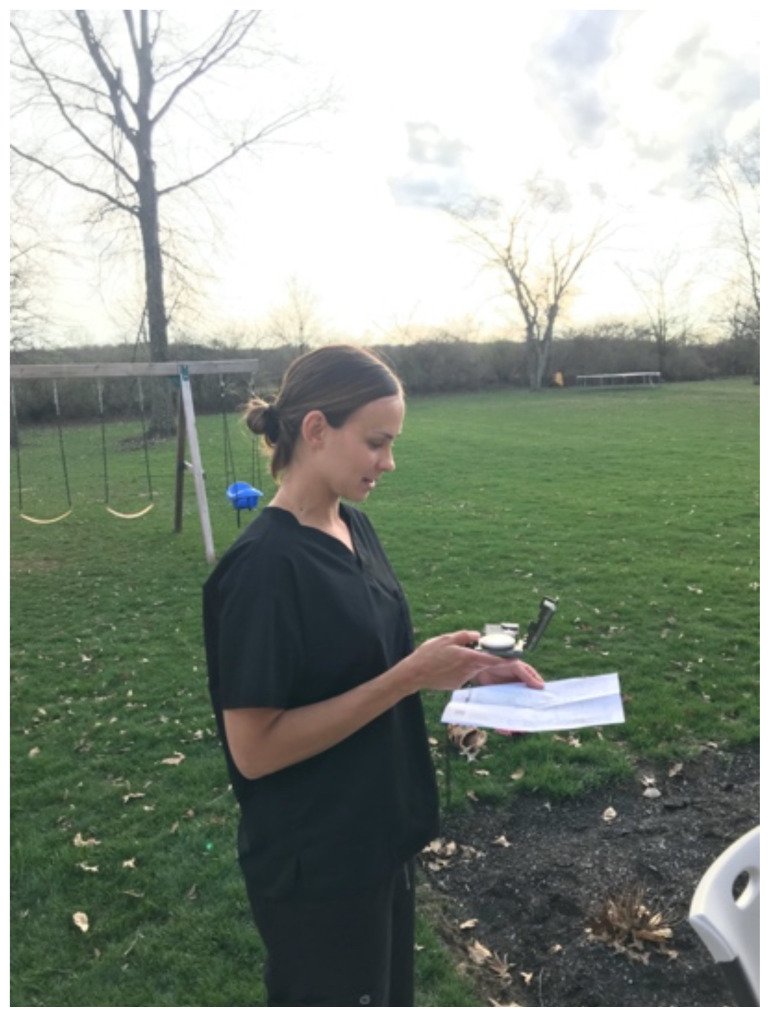
Basics of orienteering. Author’s own image

Wilderness medicine requires improvisation, the ability to provide emergent interventions and stabilization, and proficiency in teamwork which are also skills necessary for the EM provider.^1^ With an expanding interest and increasing public accessibility to outdoor recreation, there has been a documented increase in outdoor incidents requiring some form of medical attention.^2^ Emergency physicians in training should gain additional exposure to wilderness medicine knowledge, outdoor skills, and prehospital rescue techniques and risks.^3^ There has been an increase in the amount of instruction on the topic in various emergency medicine residency programs including specialty tracks and inclusion of WM topics in the educational curriculum.^4^ However, there is limited data on the instruction of general survival skills to interested residents. The main goal of this small group session was to address this need for training in the emergency medicine residency and to provide an avenue for residents with previous experience to share their knowledge. The full group of learners progressed through five survival skill stations with emphasis on improvisation, wilderness medicine knowledge, and outdoor ability (objectives 1–4). Topics included water treatment, emergency shelter creation, survival kit development, fire starting, and basics of navigation (images 1 and 2). Resident physicians from the emergency medicine residency program were invited to participate and teach in the survival event, and they were given 30 minutes to provide instruction and demonstration of a technique. They all had previous experience in the topic but were challenged to learn and develop their own expertise in order to be an effective educator. They were all provided with chapters to review from *Auerbach’s Wilderness Medicine* to assist with content, but were encouraged to find and use additional sources. Instructors were also encouraged to make the events as interactive as possible. After they finished teaching at their station, they would return to the regular group and move to the next topic as a learner participant. A debriefing occurred following the event.

This specific format was chosen with the use of multiple educational learning theories as well as focusing on the need for higher understanding and progressive teaching responsibility. Both cognitive and behavioral techniques were used by both the educators and learners to initially describe and demonstrate a skill followed by active teach-back with practice and demonstration during each station. A social learning orientation was also employed as role modeling, and behavioral rehearsal was utilized by the resident educators as they were able to impart their own recommendations and processes for each survival technique demonstration.^5^ Finally, both the educators and learners progressed through Bloom’s taxonomy starting at recalling facts all the way to being able to evaluate different skills for a single learning objective.^6^

Additionally, while the content in this small group activity is specific to those interested in wilderness medicine, the practice of learning the material, teaching the content, and applying the knowledge aligns with aspects of both the Model of the Clinical Practice of Emergency Medicine as well as the ACGME Core Competencies.^7^ Specifically, this educational experience provides the resident educator and learner with additional exposure and growth in the areas of “Interpersonal and Communication Skills,” “Professionalism,” as well as multiple items under “Practice-based Learning and Improvement.”

### Recommended pre-reading for instructor

- Backer H. Chapter 88: Field Water Disinfection. In: Auerbach PS, Cushing TA, Harris NS, eds. *Auerbach's Wilderness Medicine*. Philadelphia, PA: Elsevier; 2017:1985–2030.
- Carleton SC. Chapter 106: Wilderness Navigation Techniques. In: Auerbach PS, Cushing TA, Harris NS, eds. *Auerbach's Wilderness Medicine*. Philadelphia, PA: Elsevier; 2017:2329–2349.
- Carleton SC, Kummerfeldt P, Bowman WD. Chapter 59: Essentials of Wilderness Survival. In: Auerbach PS, Cushing TA, Harris NS, eds. *Auerbach's Wilderness Medicine*. Philadelphia, PA: Elsevier; 2017:1327–1358.
- Hovey J. Chapter 111: Nonmedical backcountry equipment for wilderness professionals.
- In: Auerbach PS, Cushing TA, Harris NS, eds. *Auerbach's Wilderness Medicine*. Philadelphia, PA: Elsevier; 2017:2409–2428.

### Learner responsible content (LRC)

- None

### Suggestions for Further Reading

- Lareau SA, Caudell MJ, Pandit KB, Hiestand BC. Medical student electives in wilderness medicine: curriculum guidelines. *Wilderness Environ Med*. 2014;25(4):474–480. doi:10.1016/j.wem.2014.04.014

### Implementation Methods

**Stations:** Many of the materials used for the course were personal property of the residents and participants. They were encouraged to bring their own supplies to assist in direct application of learning topics. The resident educators should be familiar with the location and understand the materials they will be able to procure as needed. Estimated prices are included next to the required materials in the following material details.

### Required Materials

- Water treatment and purification
  ○ Large basin of potable water
  ○ Method of halogen and filter-based treatment for demonstration (price varies based on type).
- Emergency Shelter
  ○ None. This station should be a brief discussion, and the learners should look for items in their surroundings to help build a shelter.
- Creation of a medical survival pack
  ○ None. This station should be a tabletop discussion with emphasis placed on what types of items can be used for multiple applications.
- Fire starting
  ○ Flint and steel ($2–$5)
  ○ Magnifying glass ($5)
  ○ Fire ring or area to safely start a flame
  ○ Water source for flame extinguishing
- Introduction to orienteering
  ○ Compass ($15)
  ○ Local map ($10–$20)

### Results and tips for successful implementation

This exercise is best implemented in an outdoor environment with residents of many skill levels and a specific interest in wilderness medicine. The event took place on April 8, 2019 on the property of one of the residency Wilderness Medicine/EMS interest group advisors. The event was held outside on rural farmland allowing for ample space to safely and appropriately demonstrate and practice the skills.

We formatted the evaluation as a retrospective one-sample, pre- and post-test educational evaluation. The form consisted of six questions for the participants to self-report their confidence and understanding of various survival topics (Table 1). Participants were instructed to describe their level of agreement with the statement using a ten-point scale with 10 for “Strongly Agree” to 0 for “Strongly Disagree.” There was one additional question asking if the participant found the course enjoyable, as well as a single write-in line asking for any additional comments.

For the primary analysis, we compared the total mean scores for the six items before and after the workshop utilizing the ttest. Twelve individuals participated in the WM Survival Skills Workshop and 12 (100%) participants completed the questionnaire. Twelve (100%) of the participants found the workshop to be an enjoyable experience. For the primary analysis, the total mean score for the six-item analysis increased following the workshop (39.1 before versus 51.0 after, p = 0.0008). Seven (58.3%) participants provided post-event comments (Table 2).

This course format appeared to be beneficial and enjoyable for the learner. However, implementation of the comments from the participants (Table 2) may enhance the learning experience when this small group activity is hosted again. In future course offerings, other WM topics could be interchanged with the five selected in this small group experience. Potential other topics applicable to general emergency medicine would be basics of splinting and hemorrhage control in the backcountry or low resource settings, or improvised litters or carries. These other topics may allow for a wider audience and could be tailored to include other specific components from the Clinical Practice of Emergency Medicine.

### Pearls

Water treatment and purification

- Linked objective: Differentiate at least three different methods for water purification.
- Heat, filtration, UV exposure, and various chemical disinfection techniques are all methods of water treatment.
- Weigh the benefits and limitations to each approach when selecting a method including reliability and safety.
- Water clarity is not an indication of potability, and human and animal activity is the most significant contributor of microbe pollution in surface water.^8^

Emergency Shelter

- Linked objective: Describe how to erect a temporary survival shelter.
- The specific type of shelter to build depends on specific shelter needs (degree of elemental exposure, predicted length of use), and the availability of resources.
- The basics of shelter construction include making sure there is protection from the elements. An emergency shelter must be easy to use, quick to build, durable, trap heat, and provide some protection from precipitation. Using natural features, including trees, bushes, large rocks, or snow depressions, helps decrease the number of materials needed to construct a basic lean-to or other protective shelter.^9^
- In the small group, decide the environmental location for the shelter and guide the learners to discuss needs for different climate and weather variations.
  ○ Desert, Snow, Aquatic, etc.

Creation of a medical survival pack

- Linked objective: Construct a survival pack for personal use, emphasizing multi-use items.
- There are classically 10 items needed for a survival pack: Navigation, sun protection, illumination, repair kit and tools/power, first-aid supplies, fire starter, nutrition, hydration, insulation, and emergency shelter.^10^
  ○ Ensure the learner is able to select components that will address these needs.
- Weight is key; attempt to identify items that can be used for multiple applications.
- Be prepared for specific environmental and regional challenges that may be encountered when planning for a survival kit. Type of activity, location, duration, transport to and during activity, and budget should all be considered.^10^

Fire starting

- Linked objective: Demonstrate how to make a fire without a direct flame supply.
- Three factors must be present for a fire to burn: a heat source to ignite the tinder, oxygen, and good-quality fuel.^10^ Heat sources include metal matches, flint and steel, magnifying glass/lens or others. Dry small brush, cotton balls in petroleum jelly, priming paste, or even dryer lint are all potential fuel sources for discussion.^9^,^10^
- While flame sources are easiest to use to start a fire, remember to always have a back-up that is resistant to environmental issues including wind, water, and lack of fuel.

Introduction to orienteering

- Linked objective: Recall the steps to use a compass for basic navigation and orienteering tasks.
- The process of navigation relies on the ability to establish a physical position and determination of direction. Estimation of current location is ultimately based on knowledge of the direction, rate of movement, and time of travel from a known starting point.^11^
- A magnetic compass can be used to determine cardinal directions and bearings in route finding. It can be used in conjunction with a map or through landmark spotting.^11^
- Always spend time familiarizing yourself with a map of your location prior to attempting to navigate a terrain to identify unique features, terrain variations, water, and roads.
- Practice with your compass prior to initial use in the wild.

## Figures and Tables

**Table 1:** Wilderness Medicine Survival Skills Workshop Questionnaire.

Wilderness Medicine Survival Skills Workshop
**Questionnaire**
**Items Evaluated Pre and Post Workshop**
I am aware of various water treatment options
I understand basics of emergency shelter creation
I know what types of material should be in a survival pack
I can start a fire without a direct flame supply
I know how to use a compass for basic navigation
I am confident in my personal survival skills
**Items Evaluated Post-Workshop**
Was the course an enjoyable experience? (Yes/No)
What can we do to make this course better in the future?

**Table 2:** Post-WM Workshop Comments

Post-WM Workshop Comments	
Participant 1	Build a shelter.
Participant 3	I can prepare better. Also, handouts to review over at a later date is *(**sic**)* always a positive.
Participant 4	Could consider having handout materials or more online postings in order to have access to the information from the course.
Participant 5	Break the skills into sections perhaps over multiple meetings with more hands-on practice and in-depth discussions.
Participant 7	Hands on compass course.
Participant 8	Longer sessions with more hands-on practice and materials for each to attempt the given activity.
Participant 11	Increase the available supplies for demonstration and practice.
